# Reminiscence through the Lens of Social Media

**DOI:** 10.3389/fpsyg.2016.00870

**Published:** 2016-06-14

**Authors:** Lisa Thomas, Pam Briggs

**Affiliations:** PaCT Lab, Psychology Department, Northumbria UniversityNewcastle upon Tyne, UK

**Keywords:** reminiscence, life review, Facebook, social media, slow technology

## Abstract

Reminiscence is used to support and create new social bonds and give meaning to life. Originally perceived as a preoccupation of the aged, we now recognize that reminiscence has value throughout the lifespan. Increasingly, social media can be used to both support and prompt reminiscence, with Facebook’s Lookback or Year in Review as recent examples. This work takes prompted reminiscence further, asking what forms and functions of reminiscence are supported by social media. Utilizing the online service *MySocialBook*, we invited participants to curate content from their personal Facebook account to then be transformed into a printed book. We used that book as a prompt for discussion of the reminiscence function of the curated material, using Westerhof and Bohlmeijer’s (2014) reminiscence framework as a starting point. We conclude that this framework is valuable in understanding the role of social media in reminiscence, but note that earlier models, such as Webster’s Reminiscence Functions Scale, are also relevant. We contribute to the reminiscence debate by adding a technological lens to the process of life review, whilst concurring with other researchers in this field that a robust conceptual framework is lacking, particularly when considering the forms of reminiscence that are most salient for younger people.

## Introduction

Reminiscence has most concisely been defined as *“the volitional or non-volitional act or process of recollecting memories of one’s self in the past”* (Bluck and Levine, 1998, p. 188). The first empirical studies of reminiscence focused on older adults, with the assumption that older people were more likely to engage in reminiscence in later years of life. Although reminiscence was perceived as a positive way to reconcile life events and make sense of the past, it has also been viewed as a weakness, or more specifically, an indication of cognitive decline (Westerhof et al., 2010). Early empirical studies led to the development of the life-review theory (Butler, 1963) that showed people will purposively revisit past life events, review them, and deal with any unresolved conflicts. Ultimately the review is said to help give new significance to life and increase self-esteem and satisfaction (Coleman, 1974), as well as minimize fear and anxiety about the future (Haight and Webster, 1995). However, the process of life review may also result in negative feelings of failure (Wong and Watt, 1991).

The original work on life review was very much grounded in studies of gerontology and aging, i.e., was solely seen as a valuable process for older adults. Indeed, life-review theory (Butler, 1963, 1974) was developed as a framework for understanding the role of reminiscence as a later-life act, conducted, whether conscious of it or not, in preparation for death. However, life review is now recognized as something that holds value for all ages (Tsai et al., 2013) and a considerable body of evidence shows that reminiscence, far from being a dysfunctional process, can have psychological benefits throughout the life-cycle; leading to improvements in mood, self-esteem, feelings of belongingness, and contributing to a sense of meaning in life (Sedikides et al., 2008; Routledge et al., 2013).

Reminiscence is thought to be undertaken by the young as well as old, with little difference in frequency of reminiscence (Pasupathi and Carstensen, 2003) or the emotional intensity it provokes (Westerhof et al., 2010). A formal categorization of reminiscence came from Webster (1993) who defined seven reminiscence functions that later became eight (Webster, 1997). These are captured in the Reminiscence Functions Scale (RFS), the most widely used instrument to study reminiscence and one that captures the eight functions of reminiscence as: (1) Boredom Reduction; (2) Death Preparation; (3) Identity; (4) Problem Solving; (5) Conversation; (6) Intimacy Maintenance; (7) Bitterness Revival; and (8) Teach/Inform. There have been numerous incarnations of the RFS (see Cappeliez et al., 2007; Robitaille et al., 2010) and Webster’s scale has adapted for use across a wide spectrum of age groups and cultures (e.g., O’Rourke et al., 2013 describe an Israeli sample and Ros et al., 2016 describe a Spanish sample). To a certain extent, this supports Webster and McCall’s (1999) claim that the RFS comprises a relatively universal framework for understanding the functions of reminiscence across the lifespan.

However, there has been some disagreement about the underlying model, with, for example, O’Rourke et al. (2013), Westerhof and Bohlmeijer (2014) and Ros et al. (2016) suggesting that slightly simplified three-factor models of reminiscence might yield a better fit to available data. Westerhof and Bohlmeijer (2014) describe three overarching functions of reminiscence: *social*, *instrumental*, and *integrative* (see **Table 1**). Social reminiscence reflects the ways in which people share personal memories in everyday conversations, which in turn encourages bonding, but also serves to teach others about an individual’s past experiences-something valued by older adults and indeed, something that has been shown to reduce depression in the older population (Elias et al., 2015). Instrumental reminiscence involves the remembering of previous coping strategies and applying these to current problems, such as recalling memories that might help an individual deal with bereavement. This kind of instrumental reminiscence may also help to regulate emotions when faced with current turmoil. Integrative functions, described as closest to Butler’s life review, are those that may help to cope with identity challenges during times of change. These integrative functions are also reflected in a distinct, but related literature on narrative psychology, where people use stories about their lives in order to construct a narrative identity that supports them in psychological adaptation and development (McAdams and McLean, 2013).

**Table 1:** A comparison of reminiscence taxonomies drawing from Wong and Watt’s (1991) Taxonomy of Reminiscence, Webster’s (1997) Reminiscence Functions Scale, and Westerhof and Bohlmeijer’s (2014) Functions of Reminiscence.

Reminiscence prompted by social media (current work)	Westerhof and Bohlmeijer’s (2014) Functions of Reminiscence	Webster’s (1997) Reminiscence Functions Scale	Wong and Watt’s (1991) Taxonomy of Reminiscence
**Connecting with others**	**Social:** Sharing memories in everyday conversations which fosters bonding	**Conversation:** The informal use of memories in order to connect to others **Teach/Inform:** To relay personal experiences and life lessons to others **Intimacy Maintenance:** Cognitive and emotional re-presentations of important people in our lives are resurrected in lieu of the remembered person’s physical appearance	**Transmissive:** Serves the interpretive function of passing on valued elements of one’s cultural heritage and personal legacy. **Narrative:** A descriptive rather than an interpretive recollection of the past and encompasses the sharing of routine biographical information or the recounting of past anecdotes
**Learning from the past**	**Instrumental:** Coping strategies, which by recalling past experiences, can help us learn and prepare for the future	**Problem Solving:** The use of reminiscence as a constructive coping mechanism by remembering past problem-solving strategies	**Instrumental:** Contributes to perceptions of competence and continuity and includes recollections of past plans and learning from past experience.
**Building self-knowledge**	**Integrative:** A way to reflect on the past to define one’s identity.	**Identity:** The existential use of the past to discover, clarify our sense of who we are **Bitterness Revival:** The recall of memories about unjust treatments, finding justification to maintain negative thoughts and emotions to others **Boredom Reduction:** Thinking back about the past to escape an under- stimulating environment or a lack of engagement in goal-directed activities	**Integrative:** Works to achieve a sense of self-worth, build coherence and reconciliation with the past **Obsessive:** Evidenced by statements of guilt, bitterness, and despair over one’s past **Escapist:** A tendency to glorify the past and deprecate the present, boasting of past achievements and showing a desire to return to the ‘good old days’

Technology has, for many years, played a strong role in reminiscence, for example with the use of photographs as prompts. However, a range of new digital systems now support reminiscence and life review in a range of different contexts. When away from home, newly enrolled university students report that they print off Facebook photographs and place them in their new accommodation in order to increase their sense of connectedness with family and friends (Bales and Lindley, 2013). Spontaneous, everyday reminiscence are encouraged by systems such as *Pensieve* that use social media content as triggers to provide novel reminiscence experiences (Peesapati and Schwanda, 2010). Reminiscence and life review therapy has also been developed with the aim of supporting people with diseases such as dementia (Alm et al., 2004; Huldtgren et al., 2015) or reducing depression in older adults (Elias et al., 2015). In a recent systematic review of reminiscence therapy, a number of articles outlined the benefits of technology in supporting rich and engaging reminiscence experiences (Lazar et al., 2014).

Touch interfaces and tangible objects have also been utilized as tools for reminiscence. The use of *SenseCam* as a memory aid has been shown to be beneficial for both the community (Fleck and Fitzpatrick, 2009, 2010) and for individuals (Browne et al., 2011). Other artifacts such as the *MemoryBox* (Frohlich and Murphy, 2000) have been designed to resemble the more traditional ways in which memory prompts might occupy a domestic space. The jewelry placed in this box could trigger a recorded story in audio format that could be played back to encourage reminiscence. More recently, the *Reflexive Printer* was designed to produce a grainy photograph, drawn from a social media account and destined to be deleted, thereby inducing the user to spend time reflecting on the value of the item (Tsai et al., 2014). This method encourages gradual review of digital content as a daily practice, whilst transgressing digital technology norms of media stability and quality.

Slow Technology, coined by Hallnäs and Redström (2001), is a philosophy concerned with (i) designing for slowness, solitude, and mental rest, (ii) designing interactive systems to be used across multiple generations and lifespans, and (iii) designing for slower, less consumptive lifestyles and practices (Odom et al., 2012a). Hallnäs and Redström (2001) distinguish between rapid, immediate, and visible exchanges (fast technology), and more reflective and meaningful technological experiences (slow technology). Fast technology is categorized by its ability to aid the user, making tasks faster and reducing cognitive load. In contrast, slow technology enables the opposite- increasing cognitive load, and thus deeper reflection over time. Emerging over a decade ago, it is developing as a relevant way to think about social media experiences, as well as an important consideration for designers (Beckhaus, 2012).

The slow technology theme is now being adopted as a means to deal with the morass of personal data created and stored in social media accounts. This ‘slow’ revival is in keeping with writings on the value of disconnectedness (Fullerton, 2010), and the idea that we are losing control over our lives (Leshed and Sengers, 2011) because of the constant attention technology demands of us. Research embracing the slow technology philosophy has resulted in some unique prototypes which are devised to be used over a long period of time. For example, aiming to slow down the consumption of digital photographs, *Photobox* selects images to be printed from a user’s social media account and deposits them inside a wooden chest, to imbue each with more value (Odom et al., 2012b). The ability to ‘design for pauses’ has also surfaced (Bagnara and Pozzi, 2012), reiterating the need to stop and reflect on our interactions with technologies.

The use of social media as a repository for a vast amount of information about our daily lives has become commonplace. Many people actively store valuable images and communications, with the assumption that they will remain indefinitely. As well as repositories for valued content, social media sites such as Facebook have also proliferated in recent years with methods of summarization of our digital data, playing this back to us in ‘brief automated biographies’ (Thomas and Briggs, 2015). This work identified issues with a ‘distant biographer,’ however, with content collated by others seemingly too creepy and disengaged for its viewer. Facebook’s *Year in Review*, as well as applications such as *TimeHop* now encourage us to think about our digital content that may have been long forgotten were it not for such systems. Increasingly, social media engagement is being used as a trigger for reminiscence. These processes raise the question of just how selective social media is when presenting content, and how well designed such systems are in terms of their ability to support functional reminiscence. For some time, technologies have been capable of managing the shape of our memories (Tsai et al., 2014) and new academic work in this space includes the use of lifelogging wearables to capture key moments in an individual’s day (Chowdhury et al., 2015), the use of automated biographies to capture and display personal narratives and identities (Thomas and Briggs, 2015), and the adaptation of websites such as Pinterest to curate and present material in support of inter-generational reminiscence (Brewer, 2015).

There is, however, relatively little work that documents the ways in which new digital tools offer support to different forms of reminiscence. In this paper, we explore this issue, asking what forms of reminiscence are supported by social media. We asked participants to review and edit their social media content in order for it to be displayed in a physical book that could later be used as a reminiscence prompt. We use this to assess the extent to which Westerhof and Bohlmeijer’s (2014) reminiscence functions model can be used to understand the precise value of social media in supporting reminiscence.

## Materials and Methods

### MySocialBook

This research was conducted as part of a larger EPSRC project, ‘ReelLives,’ which is investigating new ways in which people might curate their personal digital data. For this study, we needed to identify a platform that would allow both the curation and transformation of social media content into a more tangible form, suitable for later acts of reminiscence. The service *MySocialBook*^1^ was identified as a way to automatically capture life events, everyday moments, and comments from Facebook, and turn them into ‘a keepsake book.’

The study took part in two phases. Phase 1 was focused upon the editorial process and phase 2 was focused upon the reminiscence value of the finished book. It was advertised via posters, social media, university mailing lists and local organizations. We explicitly tried to involve older participants who used social media, to see if their attitudes and practices differed from younger people, something which is commonplace in other social media research (Zhao and Lindley, 2014).

#### Participants

We recruited 14 adult participants (Mean age: 41.5 years; *SD*: 14.9; range: 25–69) with an equal number of males and females. All were residents of Newcastle upon Tyne, UK, or the surrounding area. In determining numbers of participants, we were guided by the work of Guest et al. (2006) who asked ‘how many interviews are enough’ for data saturation to occur. Using data from 60 in-depth interviews, they found that saturation occurred within the first 12 interviews. Note that this study was carried out in accordance with the recommendations of Northumbria University Ethics Committee with written informed consent from all subjects. All subjects gave written informed consent in accordance with the Declaration of Helsinki.

#### Phase 1

**FIGURE 1:**
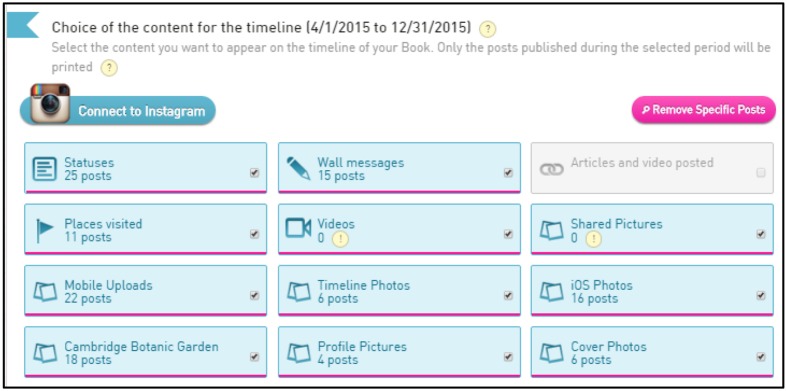
**MySocialBook curation options**.

Participants were told how they would be designing and printing their own Facebook ‘scrapbook,’ and were asked to sign into the MySocialBook site using their Facebook credentials. Participants were guided through the website with the researcher, using a laptop, being asked how they might like to design their book. The website has a number of features to encourage curation, allowing selection of different photo albums, status updates, types of posts, and shared links (**Figure 1**). Participants were asked to design their book in order to maximize the value for them. The only restriction was a price limit of £30.00 per book. Once participants were satisfied with the book they were thanked for their participation, advised it would take approximately 2 weeks to arrive by post, and informed they would be contacted by e-mail to arrange phase 2. The session lasted approximately 45 min.

#### Phase 2

**FIGURE 2:**
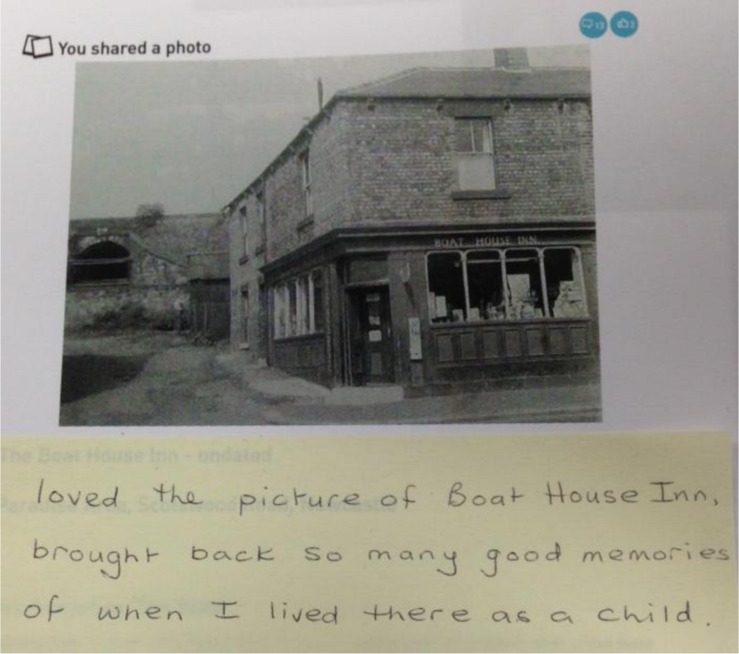
**Example of a participant’s reflection on their MySocialBook content**.

Utilizing a semi-structured interview schedule, the researcher initially asked participants what they were anticipating from their book, and what they remembered of their design choices from phase 1. Participants were then left alone in the interview room with their book, and asked to read through at their own pace. After approximately 10 min, the researcher re-joined the participant to begin discussion about their reaction to the book. Participants were asked to annotate their book with Post-its, to note anything that they liked, disliked, or thought could be improved. The results were photographed (**Figure 2**). The session lasted approximately 60 min.

### Analysis

Interview sessions were audio recorded, transcribed, and sentence-by-sentence analysis was employed using NVivo qualitative software. Initial data review was conducted by reading and rereading the transcripts for instances of actual or anticipated reminiscence. The process followed principles of thematic analysis (Braun and Clarke, 2006) as well as template analysis (see King, 2004) using an existing reminiscence framework (Westerhof and Bohlmeijer, 2014) where we examined evidence of social, instrumental, and integrative reminiscence. Note, however, that we were open to these ‘codes’ being modified in light of actual participant data. A credibility check on coding was conducted by the second author, following guidance from Elliott et al. (1999).

## Results

The data was rich in examples of actual and anticipated acts of reminiscence prompted by both the editorial process in phase 1 and the examination of the book in phase 2. Many of these themes are a good fit to the Westerhof and Bohlmeijer (2014) reminiscence framework and illustrate how it may be applied to social media contexts. We will first report on these, providing examples, before turning to themes which sit less neatly in the framework, but which resonate with other work on reminiscence and life review.

### Connecting with Others (Social Reminiscence)

The printed book was seen as a way to connect with people in the participants’ lives. When asked what they might do with it after the study, the majority of participants surmised that it would be shared with partners, parents, or friends in a social setting. Participants said they would be more likely to share this book with others, compared to reading Facebook content online which is most often a solitary activity. Our participants felt that a tangible book offered new value as something that could support explicit acts of social reminiscence:

‘*You don’t really share them too well, they’re shared in the moment and then they’ve gone (social media posts). I think the book; it could be a great way of recording your memories. Then the detail’s there, so when the family look in years to come, they’ll say, “Ah, that’s such and such, cousin whatever, with the rest of the family.” I can see it being used as a good tool’*

‘This could be a great way for families to sit together’

‘I think with a book, in all honesty, you want to talk through it with somebody else, so you’re like “Oh, look, let me show you this photo” whereas you would never do that in a million years with online Facebook’

Much of the enjoyment that participants expressed when reviewing their book was related to forgotten events or comments that featured. When this occurred, participants recognized that the book acted as a reminder for special interactions that would otherwise have been forgotten. Participants also took the opportunity for more detailed reminiscence about the specific interactions with friends and family, or expressed pleasure at being reminded of people they hadn’t heard from in a while:

‘It was my mum. I said to her “Tynemouth is awesome,” because I hadn’t been to Tynemouth before, and she’s like, “Oh that’s great,” and I said, “You need to visit, all the places are dog friendly,” and she said, “But I’m not a dog.” I was giggling about that loads. There’s things like that that just make me laugh so much that I would never have remembered that conversation if it wasn’t for this’

‘I’m working for Age UK, so I’ve started doing more Age UK notices, and that’s the women’s group I belong to. That was a conservation weekend in the Lake District. Actually I think it’s seeing people’s comments as well, it’s nice.’

The social media prompts worked well as cues for a rich social reminiscence and overwhelmingly, our participants saw this social reminiscence as a very positive thing. It is interesting, then, to note the absence of a social reminiscence component to much of the work on ‘reminiscence therapy.’ Thus, for example, in Elias et al. (2015) the integrative functions of reminiscence are highlighted as key. There is a sense, in the literature, that the subjective enjoyment of past friends is not a valued psychological function. Indeed, we could argue that this plays into a rather controversial literature on the role of social media in loneliness reduction (see Burke et al., 2010) that asserts that active and directed interaction with others is effective (e.g., leaving wall posts or messaging friends), but that the passive viewing of friends’ content only results in increased feelings of loneliness (e.g., reading but not contributing to updates and photos). We should perhaps consider the very deliberative act of social reminiscence as something distinct from these everyday, passive actions.

#### Legacy Considerations (Transmissive Reminiscence)

The legacy or ‘transmissive’ aspects of life review are very pertinent when considering social media and indeed a great deal of recent work has explicitly addressed such issues. Given our younger cohort (with a mean age of 40) we did not anticipate that social media would prompt reminiscence as a means of preparing for death and indeed, our participants did not reflect on their own mortality, *per se*. However, several did refer to their book as a way to pass on information to loved ones, something more aligned with Wong and Watt’s (1991) notion of the *transmissive* value of a life narrative:

‘I suppose once I pop my clogs, somebody could look at that and look back on it, and it’s there, but that’s the only reason I could think of it being any good to anybody’

One participant in particular was interested in family genealogy, and reflected that she felt there wasn’t any meaningful way to easily capture information about herself:

‘There’s nothing for the likes of me doing family history in 100 years’ time, for my descendants. What would there be for them to look at, to check me out and find out about me?’

Others discussed the importance of informing others of what they had achieved, or using the book as a way to learn more about family:

‘My eight stone award from Slimming World. See, I would like things like that, so that my descendants know, “Oh, she went to Slimming World, and she lost eight stone.” Do you know what I mean?’

‘I have thought about what my kids would think about my Facebook account and how little I know about my parents when they were students- how I might learn from their Facebook account sort of thing’

‘I don’t have kids, I think if you had a family of your own it would be nice to have a family one. When you got married and watching your kids grow up through to when you are retired or when your kids leave home or something. That would be a really nice family heirloom to pass down’

Such comments show how the rather trivial, day-to-day content reflected in a Facebook history sits badly with thoughts about life legacy. A recent paper on the legacy properties of social media have captured some of the contradictions around digital legacy we see here (Gulotta et al., 2013). In that study, participants saw no real legacy value in their own digital data – but paradoxically would express attachment to equivalent scrapbooks or photographs they had inherited. They quote one participant as follows: “*If my mother had an external hard drive with photographs of her entire life I would absolutely want to have a copy of these files*” (Gulotta et al., 2013, p. 1818). We can see some of these inherent contradictions here – particularly when people evaluate their social media as a form of self-presentation (see section “Trivial Content”).

### Learning from the Past (Instrumental Reminiscence)

We found relatively few examples of active instrumental reminiscence as a means of coping with the present. However, a few examples showed the value of looking back over social media content in order to remind oneself that the presence of friends could help during difficult times and that their support and encouragement could be re-experienced when re-reading posts. One participant realized that she was able to review positive comments from friends written in her book when recalling her struggle with health problems:

‘I do have a lot of health problems and that’s why I said I think I’m a bit of a miserable person, because whenever I get ill, I do get miserable. So I suppose one thing I can do now is actually look back on days where I’ve had bad days, and said something on Facebook, because there’s loads of virtual hugs. Which is quite a nice feeling’

Again, another participant noted that they had documented some unfortunate events in their life on Facebook, but the response from their Facebook audience somehow alleviated the unhappy event. These kinds of comments reflect not only the coping aspect of this instrumental theme, but also demonstrate learning from past experience:

‘I got made redundant, but looking at the book, it wasn’t negative because you look at all the responses and the comments that people made, they’re all nice and positive so it’s, yes, it was a sad time but it doesn’t look sad in the book’

Finally, comments were reflective of the ways that content in the book could provide comfort, even for a tragic event. Again, the transformed physical nature of the book as a kind of keepsake meant more than reminiscence of digital material:

‘As strange as it would sound, I think it’d be nice to see again (a friend’s death), so the comments people make, you know, so I think it would be that comfort thing. You’d keep that, you know, think highly of the person who it was, or you know, funny memories type of thing’

#### The Value of Negative Life Events

It is worth saying a little more about the way such negative events can play a role in learning from the past. Many of our participants did identify negative experiences, often associated with a relationship breakup. However, we found little evidence of bitterness tied to such negative life events. What we did find, particularly in relation to posts from former partners, were reflections on the role and prominence given to the ‘ex’ in social media. Our participants recognized that their former partner played a role in their own life story, even though they may not wish to dwell on the details of the relationship. In terms of previous frameworks, we might see that such relationships could have a role in the ‘narrative’ rather than the ‘obsessive’ forms of reminiscence (Wong and Watt, 1991):

‘There’s a comment from me posting to her (ex-girlfriend) about what she was attempting to achieve and I kind of look through that and think, “Oh, man,” that’s kind of what I wouldn’t want to have included in here, but is still a record of a relationship, I guess, and it’s littered throughout, which is – but that’s okay, because it’s worth re-visiting and having a look at’

Further, participants who were struggling to deal with a broken relationship recognized that, while they did not wish to revive unwanted feelings at this time, future acts of reminiscence might be different and indeed might change their perspective on the relationship, concluding that such ‘difficult’ content should remain accessible:

‘Say you’re a teenager at school and you’re going out with one girl and they know the ex that’s in the photograph as opposed to you’re 70 and you’ve just had a very happy 40-year marriage and we go, ‘you see this person’ and obviously they’re virtually irrelative. I think that’s interesting as well, where is that data? Do you delete it from Facebook completely or does it sit somewhere?’

Here, we get a sense of people trying to curate information for the benefit of a future self – perhaps in order to engage in some future act of reconciliation or reparation. Quite often the interviewer would ask if participants would prefer to omit certain people or events, but found that most often, people wanted to keep that information somewhere, as a record of who they are and what they have experienced. In this, we see signs of people making a rational rather than an emotional choice about curated content, something observed by Mod (2010) in their qualitative analysis of the management of relationships on Facebook, but we also see the ways in which Facebook reminiscence can be used to value the relationship differently – something observed by Zhao et al. (2012) who found evidence that couples would re-evaluate the strength or benefits of their relationship following a period of relationship reminiscence using Facebook prompts.

We should be careful, however, to avoid any conclusion that there is no role in social media for ‘bitterness revival’ (Webster, 1997) or ‘obsessive reminiscence’ (Wong and Watt, 1991). Indeed, we should be mindful of the contradictions inherent in considering Facebook or other forms of social media for reminiscence support, particularly as it pertains to relationships. Social media sites provide rich opportunities for people to deliberately construct and archive their digital selves, but they are also problematic in that they can blur the boundaries between the personal and public use of archived information. When a relationship is ‘declared’ on Facebook, a tension exists between the outward act of sharing details with friends and the inward act of maintaining autonomy, and naturally this tension can be exacerbated by a break up when negotiations around the maintenance and ownership of friends must take place (Zhao et al., 2012; Moncur et al., 2016). Sas and Whittaker (2013) proposed that disposing of digital artifacts after a break-up could be therapeutic for individuals as they move toward forming a new self-concept. However, when viewed through the lens of reminiscence, we can see that something new takes place – we are no longer talking about the public performance of self, but instead describing the private act of auto-biographical reflection. Bitterness may have a role here – but is unlikely to be one that is played out in the public forum.

### Building Self-Knowledge (Integrative Reminiscence)

The book gave participants a chance to reflect on achievements in their lives, with many of them recognizing that it provided a platform to reflect on what kind of person they were and how they might come across to others when posting on social media. For some, it was a chance to review a period of their lives that summarized a positive experience:

‘I am actually interested in why I was so happy then. When you have a good period in your life, you want to think what was so good about it? What was I reflecting? Was it just the age I was or where I was? I had those decisions in mind when I decided to do this (the study) and I think they’ve been validated actually’

As the books captured a lot of activity over a span of months and sometimes years (most people chose to review their last year), they offered a significant review of a time in a participant’s life. In some ways, reading a printed version of their transient social media posts emphasized their (online) identity all the more:

‘I’ve been told I’m really sarcastic, so I wasn’t really surprised that I came across as sarcastic, but I was surprised it came through so much’

‘I’ve always wanted to get round to printing photos off. The ones that I find particularly nice I guess I’d put on canvas, but it would be nice to capture a whole story of me. I’m not sure I’d want it from Facebook but it almost adds to my identity, well my online identity of this is me, which you almost forget’

‘You can easily imagine making one (a book) to be shared with your partner or your family and one to be maybe just shared with your parents or maybe to be shared with your children, which might include all sorts of different content and different focuses’

The above quote highlights the recognized difference between online and oﬄine identity. It also hints that one book might not be suitable for all contexts, something which aligns with the idea of more than one online identity too. The interviewer asked participants if their book provided enough information for a stranger to get a sense of who they were. Whilst most said yes, there was recognition that not everything was included. Participants were also quick to reflect on content within their book which was deemed unnecessary, often a by-product of Facebook timeline features which did not work in printed form and interfered with personal narrative. For example, one participant realized that her book included posts from a horoscope application within Facebook, and seeing them printed was annoying:

‘It’s basically just like, “This is what happened in January,” for your horoscope, it’s like, “Ooh, wonderful”’

In addition to this, we encountered numerous comments from participants about the not-so-selective nature of the application we were using, which forced them to include certain elements of their Facebook profile that they would remove if possible:

‘Take all your posts or leave all your posts. There was no, “Well, I’d like to put this bit in or I’d like to put that bit in,” so it’s a bit take it or leave it with all the tick boxes’

This formatting approach resulted in participants reviewing past experiences that they might not necessarily have chosen if the platform was more fine-tuned. This almost guaranteed that participants would experience content such as photographs of ex-partners, or people that were not consistent with their concept of identity- one participant had befriended a local councilor at some stage, who then appeared in her top friends section:

‘That one there is actually my local councilor and I hardly know him. It’s just the fact that he lives down the road from me and that’s how we communicate if I’ve got any problems with the council. I mean, how ridiculous is that?’

Thus far, we have identified examples of reminiscence present in our data which fit the framework prescribed by Westerhof and Bohlmeijer (2014). However, we also identified instances where the three broad categories did not necessarily capture the sentiments expressed in our interview data. Such themes are described below and, where appropriate, are linked to other, earlier models of reminiscence.

#### Trivial Content

People will turn to Facebook and other forms of social media when bored (Pempek et al., 2009), but this can best be seen as a form of play or distraction rather than as a form of reminiscence. While our participants did not see their printed book as a way of alleviating boredom, they did recognize the fact that some of the more trivial content had been generated as a means of boredom reduction:

‘I think like a lot of people I go on Facebook because I’m bored or frustrated about something and so you go on to vent. Even if I try to do it in a humorous way, looking back at it, I think, well, maybe a lot of that is maybe something I wouldn’t want in a book’

‘I always think, “I’d like something that’s really like funny or intellectual or relevant,” and then I never think about it, I just post, “Had a lovely day out,” and that’s it. I’m so like, “Oh, where’s my imagination?”’

Participants reflected that their books were filled with often meaningless content; posts about events or feelings which were generally unhelpful and not worth re-reading:

‘Some of this is literally organizing when I can go round to someone’s house for them to do some photos for my acting CV, so it’s like emails’

Interviewer: ‘there are 943 statuses there’

Participant: ‘Yes, but all those 943, most of them will be rubbish that I don’t want people reliving’

These reflections were not uncommon, and differentiate the purpose of social media use in the online versus oﬄine world. Participants quickly realized that although they could tailor their content to some extent, experiences that were fleeting or posts that were playful would still be archived and printed, but these posts did not necessarily enhance their reminiscence experience. Indeed, they might undermine the sense of self that was generated by social media. The act of printing Facebook statuses and photographs meant, for some, revisiting very mundane content that led them to reconsider the ways in which they might appear to others and the extent to which they took care with different forms of self-presentation.

### Serendipity

It is usual to consider reminiscence as a deliberative act, but one surprising element in our thematic analysis was the serendipitous nature of reminiscence when prompted by social media. The designing of the book resulted in an exploratory experience, where the prompts throughout led our participants down unexpected avenues. This is very different from the ways in which we might reminisce if asked directly to think about past events from memory:

‘I thought that’d be quite funny because there’ll be a mix of stuff that’s random that I don’t remember and it’s a bit serendipitous and I’d just see it and think, “That does remind me of something.” There’s a certain randomness about that and I anticipated that it would maybe make my mind go off in various directions and think, “I wonder what I was…”’

‘There’s a lot of them I had completely forgotten about, yes. I’d forgotten all about these being taken. Lovely. That was a friend of ours – we’re in Mexico but she lived in Canada, and she joined us for the holiday because it was her 60th birthday while we were there, it was lovely’

Quite often during interviews, participants would begin by talking through the events that were depicted in their book, actively enjoying the recollection of what they had done. Despite the interviews taking place at a time when social media platforms were encouraging people to make videos of their experiences and ‘look back’ on past content, participants welcomed the opportunity to review content as if it hadn’t been seen for a long time. Perhaps there was also something unique in the evocative power of the source material itself. When asked, unprompted, to review a life, we tend to behave in rather predictable ways, citing major life events such as a first day at school, a marriage, or the birth of a child – drawing more heavily upon a particular period in life – known as the ‘reminiscence bump’ (Holmes and Conway, 1999). However, we can be induced to recall other elements of the life script in order to engage in richer forms of reminiscence (Ece and Gülgöz, 2014) and it seems as though the scrap-book of social media may have additional value here.

## Discussion

This study has investigated the role of transformed or ‘slow’ social media as a reminiscence tool for both young and old. Responding to van den Hoven’s (2014) observation that “*Digital media, consisting of bits and bytes, are just not as visible and present as physical or tangible media are*” (p. 370), we have engaged with participants to create printed books from their social media profiles and assessed the reminiscence value of such books. Participant responses were measured against existing theoretical frameworks regarding the role and function of reminiscence. Our data generally supported the three overarching reminiscence categories described by Westerhof and Bohlmeijer (2014) which we have described in terms of the three acts of connecting with others, learning from the past and building self-knowledge, although we should note that our examples of learning from the past (instrumental strategies) were rather limited.

Other reminiscence functions (taken from the earlier models of Wong and Watt, 1991 and Webster, 1997) have been discussed as clustered within these three categories. For example, participants were willing to discuss negative life events, particularly around relationship break ups, death, or employment problems, but recognized that these currently provided subjects for reminiscence only in the sense of having a full life narrative available. However, participants also recognized that such material provided possible triggers for future acts of reparation and reconciliation, i.e., they were able to see themselves as resilient individuals who would ultimately be able to cope with bitterness. Our participants considered this material as worth keeping for some future time in which it might provoke a more profound reminiscence on the ways in which they have dealt with life’s difficulties (cf. the relationship work of Zhao et al., 2012; Moncur et al., 2016). This same kind of future focus was evidenced when considering the legacy or transmissive (Wong and Watt, 1991) value of a book about one’s life that could be shared with others in the event of death. Again, we note that our participants were younger than those most commonly recruited to studies on reminiscence and life review and we may have observed more legacy concerns in an older population (Thomas and Briggs, 2014).

The inclusion of trivia in the book (typically an artifact of posts previously made for entertainment or boredom reduction purposes) presented participants with unexpected but sometimes highly effective prompts for reminiscence. These sample postings served two main purposes – either as a means of disparaging the self (‘I’m so shallow’) which could be considered as an ‘integrative’ function in life review, or as a means of triggering unusual memories that would otherwise have lain dormant. The latter is interesting when we consider that such memory cues have never really been a part of our cultural make up before now. Earlier, we noted a paradox in the value ascribed to social media data. We perceive such trivial, day-to-day information as worthless, yet when we inherit such material, e.g., in the form of a scrapbook that belonged to a deceased family member, we become highly attached to it (Gulotta et al., 2013). We think there is a need to understand more about the potential future value of daily social media fragments as memory cues that could move us away from a reliance on milestones such as weddings and birthdays. We have a new and unique opportunity to compare the reminiscence power of traditional, purposefully curated prompts (such as a wedding album) with the mundane content of a daily social media profile. The latter would probably be discarded as valueless by many, but could represent the kind of content that could release long-hidden memories in older life or that could hold real value for others, who are often the people tasked with sorting legacy objects left behind (Gulotta et al., 2013).

Our findings suggest that we need to understand more about how the reminiscence value of social media content may change over time. We have seen this with two very different examples: firstly in relation to negative or unpleasant content such as a relationship break-up, where participants are reluctant to delete material in part because it was part of their history but also because it may offer future opportunities for reparation. Secondly, in relation to trivial items, considered valueless at present, but that could serve as a future triggers for otherwise hidden memories. In designing for reminiscence, these issues are important, but under-researched. We recognize that some recent work on slow media has shown the value of an enforced time-delay on social media posting that results in a more ‘profound’ form of reminiscence (Odom, 2015) and so we might speculate on the design value of a ‘hidden chest’ for unwanted or upsetting material that sits unseen but could be reviewed at some later point. We would also like to see a richer consideration of the value of digital data in reminiscence therapy. At present, it is common for prompts such as photographs, images, or music from the past to be used in such therapies – either to support the individual in accepting past conflicts (integrative reminiscence therapy) or to elicit successful means of dealing with difficult situations in the past (instrumental reminiscence therapy). We have seen here that social media, if well managed, has the potential to offer a wider range of effective cues to recall and yet we still know very little about the power of these different cues to trigger wellbeing changes.

In summary, we feel we have made three distinct contributions to the field of reminiscence research. Firstly, we have mapped the ways in which social media can be used for reminiscence and have begun the process of mapping the different forms of reminiscence that can be elicited by social media onto existing models. Secondly, we have explored reminiscence for a wider range of participants than is typical (given its widespread use in gerontology). We have shown that a social-media fueled process of life review and reminiscence can hold benefit for younger as well as older people. Finally, we have touched on some of the design issues in this space, noting the value of ‘slow media’ and briefly considering the ways form may drive function. We have noted the ways in which people may plan for reminiscence later in life and would recommend more design research aimed at supporting such planning processes. Here, we might also note that while the research and design literature around digital legacy is growing, there is no accompanying work on planning for life review.

The act of designing the book from their own social media account was exciting for participants, and often the reaction when they got to review their book was of overwhelming satisfaction and enjoyment – those participants who were initially cynical about the simplicity of printing Facebook content explicitly talked of their surprise at how good it looked, or how much they enjoyed revisiting their online experiences in printed form. These sentiments were often accompanied with the realization that without printing their Facebook content, they would not have recalled the activities they posted about online at any other time. The books encouraged participants to think about sharing their experiences with loved ones, compared to the online review of Facebook which was something people said they were most likely to do alone, if at all.

Using social media as a reminiscence tool was not unproblematic. Participants struggled to manage vast quantities of data and felt that the website offered them a fairly restricted set of tools with which to create their book. Many participants said they would prefer better editorial control and that they would gladly spend the time to make the book a truly tailored artifact. Our participants recognized that the quality of the finished books was dependent upon the quality of their social media profile – i.e., some life events were absent from their Facebook accounts and therefore unavailable for inclusion. However, this approach was a valuable first step to understanding if and how people may utilize and potentially augment social media as part of a process of reminiscence and life review.

## Author Contributions

LT and PB worked together on this research, and jointly analyzed the data to inform this submission’s findings. The manuscript was written with input from both LT and PB.

## Conflict of Interest Statement

The authors declare that the research was conducted in the absence of any commercial or financial relationships that could be construed as a potential conflict of interest.

## Footnotes

1 https://www.mysocialbook.com/
